# Metronomic Dosing for Pancreatic Cancer Combination Therapy Treatment Association With Reduced Neutropenia

**DOI:** 10.1200/GO.22.22000

**Published:** 2022-05-05

**Authors:** Sharoon Gill, Rakesh Killian

**Affiliations:** ^1^ Brooklyn Cancer Center, Brooklyn, New York

## Abstract

Metronomic dosing for pancreatic cancer combination therapy treatment association with reduced neutropenia.

**METHODS:**

This research aims to evaluate the use of combination therapy for shrinking tumor size in patients with metastatic pancreatic cancer using gemzar/paclitaxel combination therapy for treatment. Patients with pancreatic cancer were treated and observed in a cancer outpatient clinic. Gemzar/Paclitaxel combination therapy was used in lower than recommended dose. Patients were educated on the risk of neutropenia associated with combination chemotherapy. Labs were repeated every month to assess the tumor size, metastasis of tumor growth to other organs and neutropenia associated with combination chemotherapy.

**RESULTS:**

Patient in the clinic undergoing chemotherapy showed that using gemzar/paclitaxel combination therapy has reduced chance of neutropenia when used with low dose intervals in metastatic pancreatic cancer.

**CONCLUSION:**

We observed that using metronomic dosing in patients using combination therapy for pancreatic cancer shows decreased neutropenia compared to others who did not receive metronomic dosing on combination treatment with gemzar/paclitaxel. While neutropenia is decreased, patients also have decline in the metastasis of the tumor to other organs and shrinkage in the size of tumor.

